# The Secretory Apparatus of *Tabernaemontana ventricosa* Hochst. ex A.DC. (Apocynaceae): Laticifer Identification, Characterization and Distribution

**DOI:** 10.3390/plants9060686

**Published:** 2020-05-28

**Authors:** Clarissa Naidoo, Yougasphree Naidoo, Yaser Hassan Dewir

**Affiliations:** 1 School of Life Sciences, University of KwaZulu–Natal, Westville Campus, Private Bag X54001, Durban 4000, South Africa; naidooclarissa5@gmail.com; 2 Plant Production Department, P.O. Box 2460, College of Food & Agriculture Sciences, King Saud University, Riyadh 11451, Saudi Arabia; ydewir@ksu.edu.sa; 3 Department of Horticulture, Faculty of Agriculture, Kafrelsheikh University, Kafr El-Sheikh 33516, Egypt

**Keywords:** laticifers, latex, articulated, anastomosing, alkaloids

## Abstract

Due to the inconsistencies in the interpretation of laticifers within the Apocynaceae, the current study aimed to distinguish, for the first time, the type and distribution of the laticifers in the embryos, seedlings and adult plants of *Tabernaemontana ventricosa* (Forest Toad tree). The characterization and distribution of laticifers were determined using light and electron microscopy. The findings revealed the presence of articulated anastomosing laticifers. The laticifers were found to have originated from ground meristematic and procambium cells and were randomly distributed in all ground and vascular tissue, displaying complex branching conformations. The presence of chemical constituents within the laticifers and latex determined by histochemical analysis revealed the presence of alkaloids, phenolics, neutral lipids, terpenoids, mucilage, pectin, resin acids, carboxylated polysaccharides, lipophilic, and hydrophilic substances and proteins. These secondary metabolites perform an indispensable role in preventing herbivory, hindering and deterring micro-organisms and may possibly have medicinal importance. The outcomes of the present study outlined the first micromorphology, anatomy, ultrastructural and chemical analysis of the laticifers of *T. ventricosa*. In addition, this investigation similarly established the probable functions of latex and laticifers.

## 1. Introduction

The occurrence of laticifers and latex has been observed in approximately 12,500 plant species representing 22 families [1,2,3]. Latex is characterized as a sticky suspension of several particles containing a sap of various plant metabolites [3,4,5]. These naturally occurring secondary metabolites are formed from several constituents which are usually produced via primary and secondary metabolism [6,7]. Plant secondary metabolites can be divided into three chemically distinct groups: Terpenoids, phenolics, and nitrogen/sulfur containing compounds [8,9,10]. These diverse secondary metabolites are essential for plant growth, development, interactions, and defense systems [6,7,8,9,10].

Natural secondary products such as latex are comprised of a variety of composite chemical constituents which is often species dependent for, e.g., terpenoids (*Hevea brasiliensis*), alkaloids (*Papaver somniferum*), phenolic glucosides (*Cannabis sativa*), proteins (*Ficus callosa*), and tannins (*Musa* sp.) [11]. The occurrence of these chemical constituents could attribute to the appearance of latex as the color varies in plant species and may appear milky white, yellow, orange, red, brown, or even colorless [12,13]. Despite, the extensive occurrence of latex bearing plants in approximately 10% of flowering species, there remains insufficient information available concerning the characterization, distribution, and secretory mechanisms of laticifers in numerous plants [1,2,3].

Laticifers are defined as highly specialized cells (or files of cells) and have been classified into two types: The articulated type and the non-articulated type [14,15,16,17]. Articulated laticifers occur in both the primary and secondary bodies of a plant and are comprised of multiple interconnected simple cells [13,14,15,16,17]. These cells are distinguished as either nonanastomosing (unbranched) or anastomosing (branched) [2,3]. Whereas, non-articulated laticifers usually only arise in the primary bodies of plants, are multinucleate and develop from a single coenocytic cell [13,14,16,18]. These cells may display one of two forms: Unbranched vessels or branched networks [14,19]. 

It has been reported that non-articulated laticifers are an ancestral characteristic feature of the Apocynaceae, which often occur in all vegetative and reproductive organs of the family; however, articulated laticifers have also been observed in *Tabernaemontana catharinensis* [2,3,20,21]. *Tabernaemontana ventricosa* Hochst. ex A.DC. [22], belongs to the Apocynaceae and has a disjunctive geographical distribution in Eastern Nigeria, Ghana, the Democratic Republic of Congo, Kenya, and the northern and southern regions of South Africa [23]. The species occurs in open forests and thickets in woodlands and thrives in disturbed shady habitats [24]. In KwaZulu-Natal, the bark, stems and leaves of *T. ventricosa* are used in ethnomedicine to palliate fever, reduce high blood pressure, treat wounds and heal sore eyes [22,23,24,25]. Limited studies are available on *T. ventricosa* [22,23,24,25], and there are no existing reports on the morphology, anatomy, histochemistry, and ultrastructure of the laticifers of this species. 

Due to the challenging interpretation and uncertainties of laticifers in Apocynaeceae, this study was carried out to describe the type and distribution of laticifers in embryos, seedlings and adult plants of *T. ventricosa*, as well perform a chemical analysis of the laticifers and latex to identify its probable functions. 

## 2. Results and Discussion

### 2.1. Leaf Micromorphology

**Figure 1:**
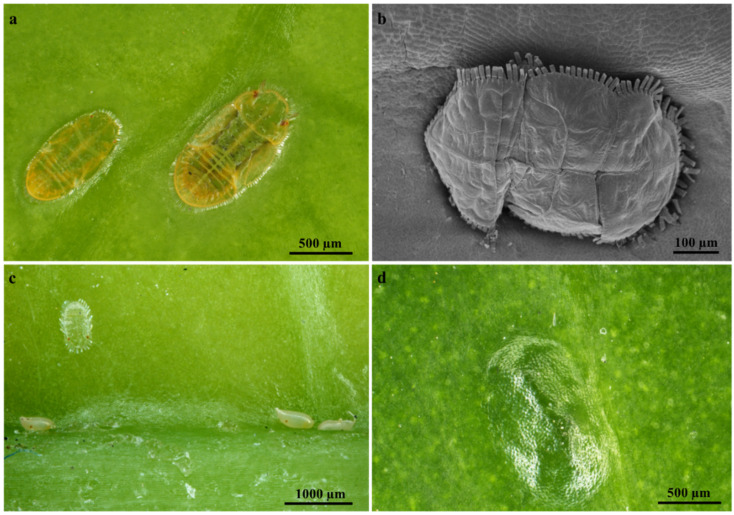
Micrographs showing insect-herbivore interactions on *T. ventricosa* adult plant leaves. (**a**) Mites appear embedded on the glabrous adaxial leaf surface of an emergent leaf. (**b**) Scanning electron microscopy micrograph showing a high-magnification image of an embedded mite on the adaxial leaf surface of an emergent leaf. (**c**) Stereomicrograph showing a waxy cuticle layer, an embedded mite and mite pupa on the shiny abaxial surface of a young leaf. (**d**) Sunken depression (Imprint from mite) present on the leathery and shiny adaxial surface of a mature leaf.

Stereomicrographs revealed that the leaves of *T. ventricosa* display a glabrous, leathery, and shiny adaxial and abaxial leaf surfaces with no prominent leaf structures (Figure 1a–d). The analysis of the leaf morphology of *T. ventricosa* is consistent with previous botanical observations [23]. Interestingly, there were occurrences of mite mastication on the waxy surface layer of the emergent leaves (Figure 1a–c).

Mites were observed to be embedded on the adaxial leaf surface of emergent and young leaves. There were no observations of mites being present on the adaxial surfaces of mature leaves; however, the manifestation of mites embedded on emergent leaves resulted in the formation of depressions (imprints) on the adaxial leaf surface of mature leaves (Figure 1d). According to these results, it is exceedingly probable that herbivory has a crucial effect on the development of vegetative organs and the chemical composition of *T. ventricosa* [23,26]. Moreover, it has been suggested that the chemical composition within leaves are altered by insect-herbivores such as mites which rapidly feeds off latex [16,26], which latex is effective against chewing herbivores [26,27]. Therefore, the mites may be possibly challenged by the high concentrations of latex defense substances present within mature leaves [26,27,28]. This chemical defense trait plays an essential role in protecting plants against herbivore attacks [16,26,28]. The most probable function of laticifers is protection [15,16,26,27], and this is evident in our findings on the laticifers of *T. ventricosa*.

### 2.2. Ontogeny and Structure of Laticifers

**Figure 2:**
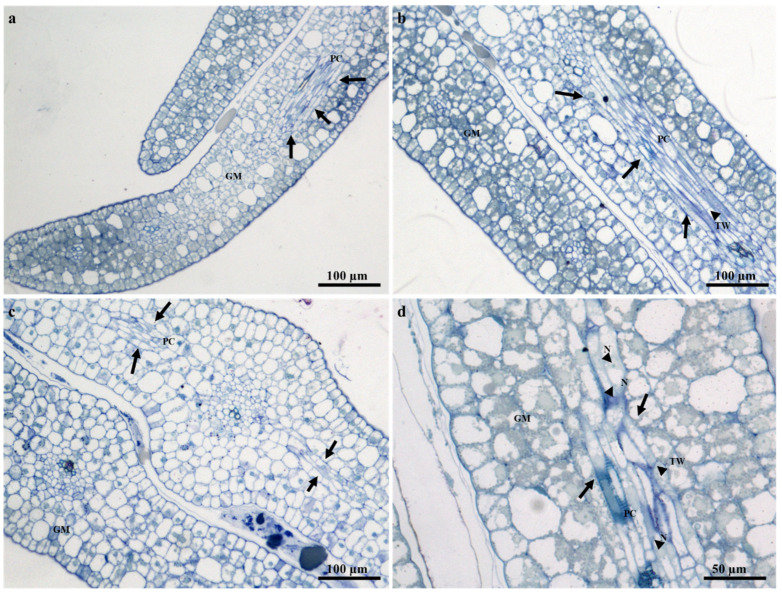
Light-micrographs showing the anatomy of laticifers in the mature embryos of *T. ventricosa*. (**a**) A longitudinal section through the mature embryo displaying the formation of articulated laticifers in cotyledons. (**b**) A longitudinal section through the mature embryo depicts the arrangement of articulated laticifers in the vascular region. Note the terminal walls within the cell. (**c**) A transverse section through the embryo displaying the occurrence of laticifers in the hypocotyl region. (**d**) A transverse section showing a highly magnified region of vascular tissue and laticifers in the hypocotyl area. Note the occurrence of terminal walls, and multinucleated laticifers Abbreviations: TW = terminal wall, N = nucleus, GM = ground meristem, PC = procambium. Arrows refer to laticifers.

**Figure 3:**
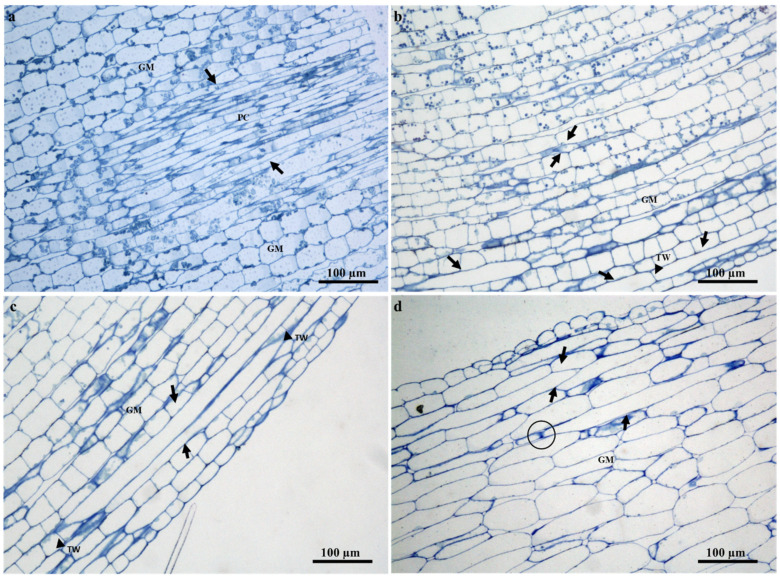
Light-micrographs showing the anatomy of laticifers in the seedling stem of *T. ventricosa*. (**a**) A longitudinal section through a seedling stem displaying the occurrence of articulated anastomosing laticifers. (**b**) A longitudinal section through the stem of a seedling depicts the arrangement of articulated laticifers closely associated with the epidermal tissue. (**c**) A transverse section through the seedling stem displaying an elongated-tapered syncytia cell, with terminal walls at the lateral ends. (**d**) A transverse section through the seedling stem showing the arrangement of overlapping laticifers. Note the cell wall dissolution of terminal walls at the tapered regions. Abbreviations: TW = terminal wall, GM = ground meristem, PC = procambium. Arrows refer to laticifers. Circle depicts cell wall dissolution.

**Figure 4:**
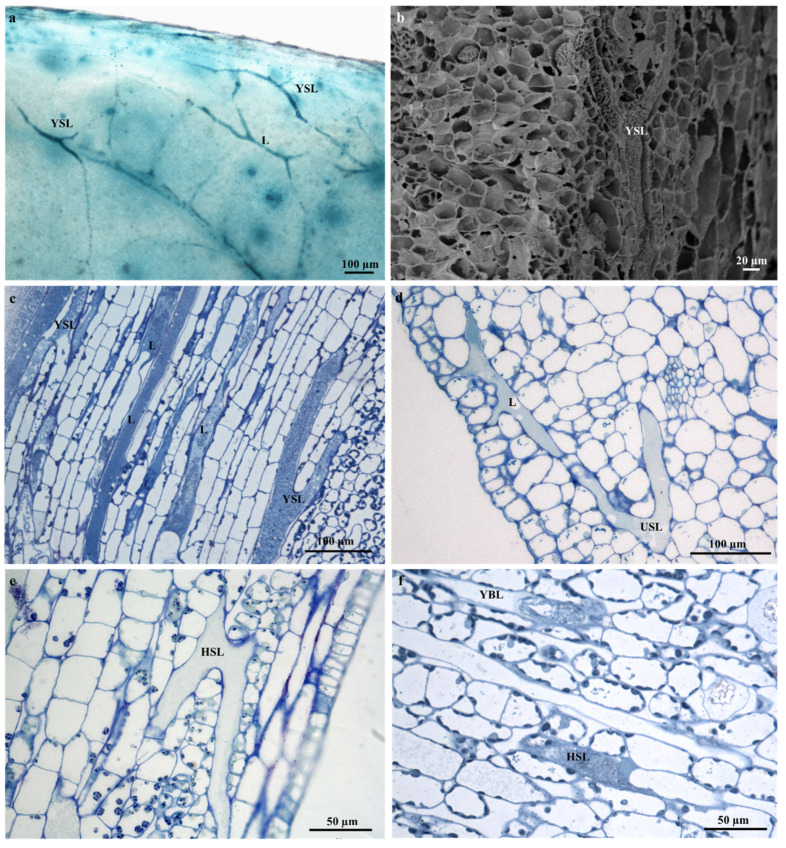
Articulated anastomosing laticifers of *T. ventricosa*. (**a**) Light micrograph of a whole stained seedling leaf showing a branched network of Y-shaped laticifers. (**b**) Scanning electron microscopy micrograph of a young stem fracture showing a single Y-shaped branched laticifer. (**c**) Light micrograph of sequential sectioned young leaf showing Y-shaped laticifers (**d**) Light micrograph of an emergent leaf section showing a branching laticifer and a U-shaped branched laticifer. (**e**) Light micrograph of a young stem section displaying a branched H-shaped laticifer. (**f**) Light micrograph of a young stem section depicts branched Y- and H-shaped laticifers. Abbreviations: L = laticifer, USL = U-shaped laticifer, YSL = Y-shaped laticifer, HSL = H-shaped laticifer.

The laticifers of several species within Apocynaceae are often recognized as nonarticulated; however, articulated laticifers have been observed in a few species [20,29,30,31,32]. Articulated laticifers are described as multiple interconnected simple cells, with intact or perforated transverse walls and lateral walls [16,18,20,26]. By analogy, the present study reports for the first time the presence of articulated anastomosing laticifers in the embryos, seedlings, and adult plants of *T. ventricosa* (Figure 2, Figure 3 and Figure 4). These results are relatively confounding with previous reports [29,30], and simultaneously consistent with current reports that have described the presence of articulated anastomosing laticifers in Apocynaceae [18].

**Figure 5:**
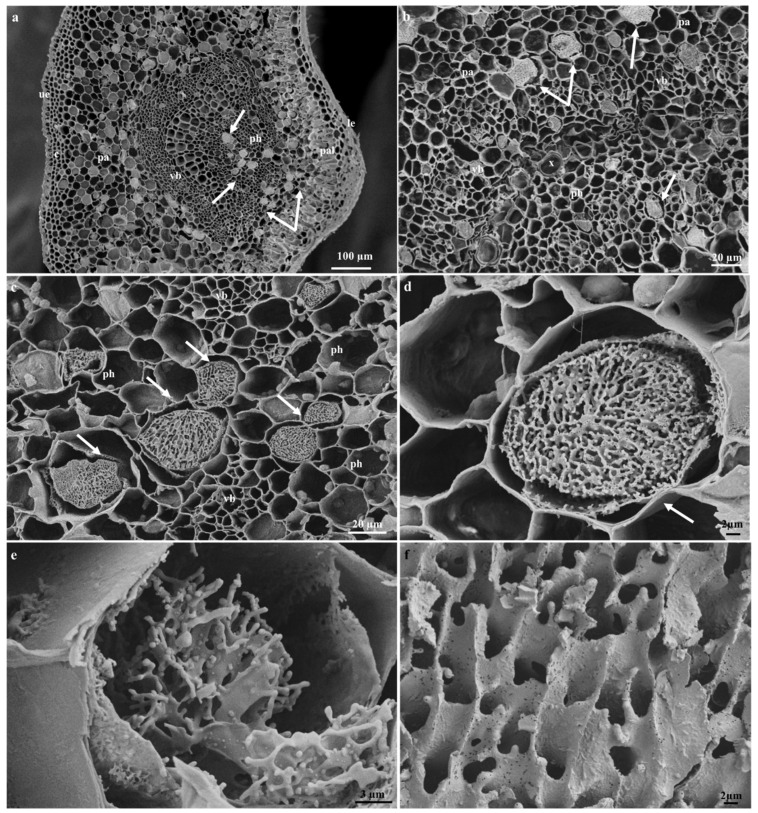
Scanning electron microscopy micrographs showing freeze-fractures of the adult leaves of *T. ventricosa*. (**a**) Low-magnification SEM micrograph showing a freeze-fracture of the midrib from a young leaf. (**b**) Freeze-fracture showing the distribution of laticiferous cells along the vascular bundles and phloem of a mature leaf midrib. (**c**) Laticifer cell distribution among vascular bundles of a young leaf midrib. (**d**) Laticifer cell showing latex exudate from a young leaf. (**e**) Latex exudate within laticifer cell from a young leaf. (**f**) High-magnification image showing the appearance of latex exudate from an emergent leaf. Abbreviations: ue = upper epidermis, c = collenchyma, pa = parenchyma, vb = vascular bundles, ph = phloem, x = xylem, pal = palisade, le = lower epidermis. Arrows refer to laticifer.

The prevalence of articulated anastomosing laticifers which is novel for *T. ventricosa* have been classified through rigorous analyses of mature embryos and seedlings, which is recognized as an essential technique to establish the development and characterization of laticifers [14,18,31,33]. The analyses of embryos (Figure 2) and seedlings (Figure 3) showed that the laticifers of *T. ventricosa* were found to have originated from ground meristematic tissue and procambium cells. Whereas, in the adult plants the laticifers were initiated from the vascular tissue (Figure 5b), comparable to reports by Canaveze and Machado [18]. The progression of laticifers were observed to occur in the primary embryonic stages (Figure 2). These processes are simply notable due to distinctive characteristics such as thickened lateral walls, axial elongations, multinucleated spherical nucleus (Figure 2d), terminal walls (Figure 2b,d and Figure 3b,c), and granular-filled protoplast (Figure 2d). In the seedlings (Figure 3c,d), lateral longitudinal laticifers are closely associated and were found to display an acute apex. According to Gama et al. (2017), these observations are the outcome of oblique sectioning of the laticifer apex [31]. Besides, there is no consistent indication of intrusive growth around the surrounding tissues of the laticifer apex (Figure 3).

The laticifer system of *T. ventricosa* embryos are composed of straight and narrow laticifers (Figure 2), whereas sinuous and wide laticifers are observed in the seedling (Figure 3), the adult plant leaf blade and young stem sections (Figure 4), similarly to other studies [2,3,18,21,31,32,33]. The continuous branching of laticifers usually results in the formation of a network comprised of multiple laticifers that expand and connect throughout the primary and secondary bodies of the plant [31,32,33]. Laticifers are habitually accompanied by meristematic and adjacent cells that develop into an anastomosing complex system comprised of “Y”, “H”, or “U”conformations [31,32,33,34]. In Figure 4a–f, distinctive branching conformations are seen, which indicate that the laticifer branching patterns in the adult leaves and stems of *T. ventricosa* are consistent with those observed in the genus [18,21].

### 2.3. Distribution of Laticifers

**Figure 6:**
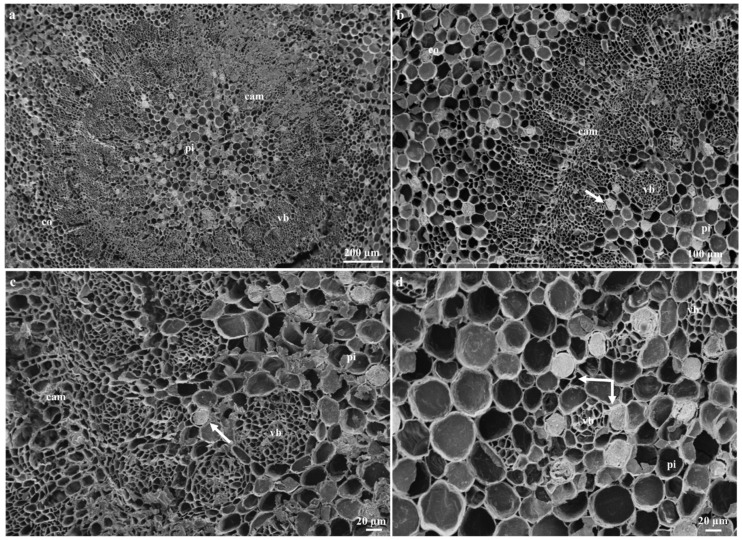
Scanning electron microscopy micrographs showing freeze-fractures of adult stems of *T. ventricosa.* (**a**) Low-magnification SEM micrograph showing the distribution of laticifers in a freeze-fracture of a young stem. (**b**) Freeze-fracture of a young stem depicts the arrangement of laticiferous cells along the vascular cambium. (**c**) Laticifer cell distribution among vascular bundles of a young stem. (**d**) High-magnification image showing appearance of laticifer and latex exudate from a young stem. Abbreviations vb = vascular bundles, cam = cambium, co = cortex, pi = pith. Arrows refer to laticifer. Arrows refer to laticifers.

**Figure 7:**
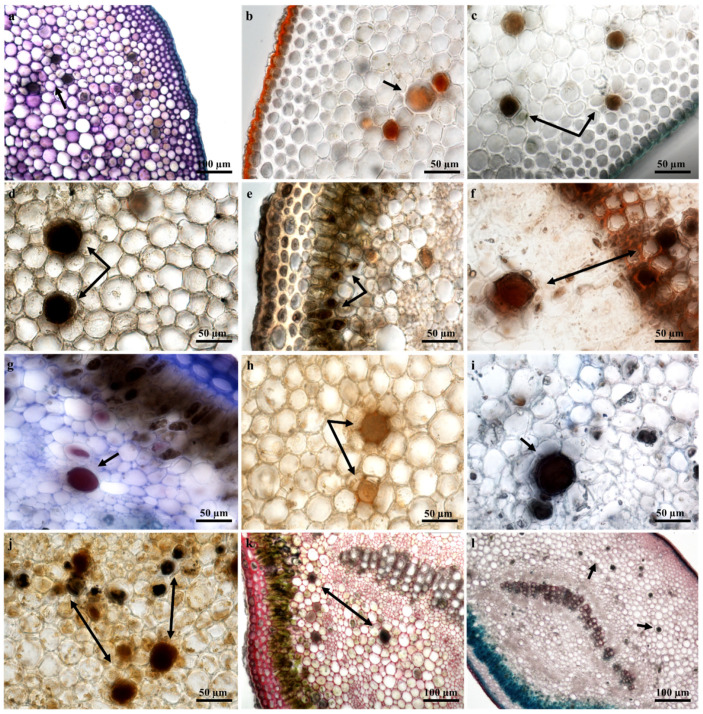
Histochemical observations of laticifers in young leaf midrib sections of *T. ventricosa*. (**a**) Presence of polysaccharides within laticifer cells stained using Toluidine Blue. (**b**) Positive staining of lipids within laticifers using Sudan IV. (**c**) Negative staining of lipids, cutin, and suberized cell walls of laticifers using Sudan black B. (**d**) Ferric Trichloride positively stained laticifers a dark-black color. (**e**) Presence of phenolics within laticifer cells stained using Ferric Chloride. (**f**) Intense staining of resin acids within laticifers using NADI reagent. (**g**) Intense staining of neutral lipids within laticifers using Nile Blue. (**h**) Negative staining of lignin aldehydes within laticifer and cell components stained using Phloroglucinol. (**i**) Intense blue-black staining of proteins in laticifers. (**j**) Intense staining of alkaloids within laticifers stained using Wagner’s and Dittmar’s reagent. (**k**) Positive staining of mucilage and pectin using Ruthenium Red. (**l**) Presence of acidic substances in laticifers stained using Safranin and Fast Green. Arrows refer to laticifer.

Articulated anastomosing laticifers are present in all ground and vascular tissues and are closely associated with the phloem of young and mature leaf sections (Figure 5a–c). In addition, laticifers were found scattered in the mesophyll region and often extend towards the leaf extremities (Figure 4a,c,d and Figure 7a–c). These results confirm previous findings on growth mode and development of laticifers in *Tabernaemontana catharinensis* [18,21] and *Allamanda blanchetii* [31]. In the stem, laticifers occur in the cambial region, cortical parenchyma, vascular tissue, and pith (Figure 6a–d). In plants laticifers often display extensive distribution patterns [33,35,36]. However, in some instances, the location may differ within vegetative organs, as it is assumed that the distribution patterns of laticiferous cells may possibly be species-specific and might result as a valuable tool for classification at a taxonomic level [33]. These distribution patterns are relative to those seen in the genus *Tabernaemontana* [2,3,18,21,31,32,33].

### 2.4. Laticifer Histochemical Characterization

**Figure 8:**
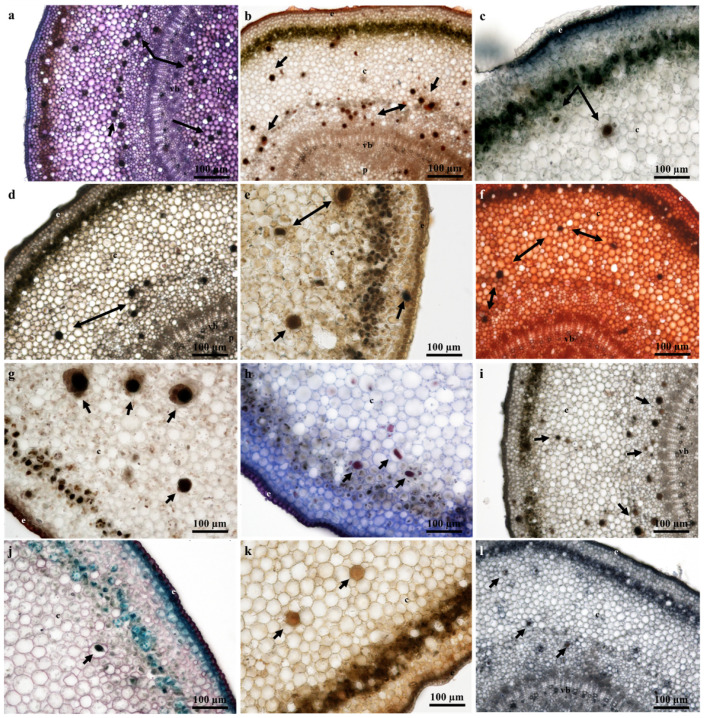
Histochemical observations of laticifers in young stem sections of *T. ventricosa*. (**a**) Presence of polysaccharides in laticifers stained using Toluidine Blue. (**b**) Lipids stained using Sudan IV. (**c**) Negative staining of lipids using Sudan Black B. (**d**) Ferric Trichloride positively stained laticifers a dark-black color. (**e**) Alkaloids identified within laticifers using Wagner’s and Dittmar’s reagent. (**f**) Detection of mucilage and pectin using Ruthenium Red. (**g**) Intense staining of resin acids in laticifers using NADI reagent. (**h**) Neutral lipids in laticifer identified using Nile Blue. (**i**) Detection of phenolics within laticifer stained using Ferric Chloride. (**j**) Presence of acidic substances within laticifers stained using Safranin and Fast Green. (**k**) Negative staining of lignin aldehydes using Phloroglucinol. (**l**) Proteins detected in laticifers. Arrows refer to laticifer.

The milky white latex of *T. ventricosa* is a synapomorphy of the Apocynaceae [13,37,38,39]. Considering the composite secretion of latex which is often comprised of a variety of specialized metabolites, several latex bearing plants are well-known for their specific substances [29,33,40,41]. The histochemical analysis of the laticifers and latex within the vegetative organs of *T. ventricosa* revealed for the first time in this species the presence of carboxylated polysaccharides (Figure 7a and Figure 8a), lipophilic and hydrophilic substances (Figure 7b and Figure 8b), proteins (Figure 7i and Figure 8l), phenolics (Figure 7e and Figure 8i), terpenoids (Figure 7d and Figure 8d), neutral lipids (Figure 7g and Figure 8h), alkaloids (Figure 7j and Figure 8e), mucilage and pectin (Figure 7k and Figure 8f), resin acids (Figure 7f and Figure 8g), and acidic substances (Figure 7l and Figure 8j). These findings are consistent with previous studies, as the compounds within the laticifers and latex were previously detected in species belonging to the genus *Tabernaemontana* and may be of therapeutic and pharmacological importance [42,43,44,45,46,47]. Furthermore, regarding the chemical constituents detected within leaf and stem sections of *T. ventricosa*, studies have shown a possible association between the secondary metabolites of latex and defense mechanisms against pathogens and herbivores [13,15,16,20,27,48,49]. The latex (Figure 5d–f) found within the laticifers of *T. ventricosa* contains chemical compounds (Figure 7a–l and Figure 8a–l), which may be lethal or function as a preventative towards herbivores and pathogens and most likely hinder micro-organism proliferation [13,16,26,27,33,46].

A positive and intense color reaction for alkaloids using Wagner’s and Dittmar’s reagents was observed in the laticifer protoplast of the vegetative organs (Figure 7j and Figure 8e). These results are consistent with the first phytochemical investigation performed on *T. ventricosa* as major alkaloidal components namely, 10-hydroxyheyneanine and akuammicine were detected and isolated from the leaves and stembark [46]. According to the results of the current study, the traditional usage of the plant may possibly be related to the high presence of alkaloids, which is often used for the treatment of various ailments such as high fever, pain, and exposed wounds [23,25]. Moreover, previous reports on *Tabernaemontana* species showed that the alkaloids dregamine and voacangine isolated from *T. elegans* and indole alkaloids found in *T. catharinensis* exhibited substantial anti-bacterial activity and have medicinal value [47,50]. It has been observed that plants within the genus *Tabernaemontana* often obtain a profusely high alkaloid content, usually displaying biological activity [42,45]. According to Van Beek et al. (1984), alkaloids are organic nitrogenous compounds with the nitrogen being either in its primary, secondary, or tertiary form [43]. Additionally, monoterpene indole and bisindole alkaloids are the major classes of alkaloids within the *Tabernaemontana* genus, other compounds include terpenes, lactones, steroids, phenolics, and flavonoids [42,43,44,45]. A few of the latter compounds, namely alkaloids (Figure 7j and Figure 8e), terpenoids (Figure 7d and Figure 8d) and phenolics (Figure 7e and Figure 8i) have been observed in the current study. 

#### Laticifer Fluorescence Microscopy

**Figure 9:**
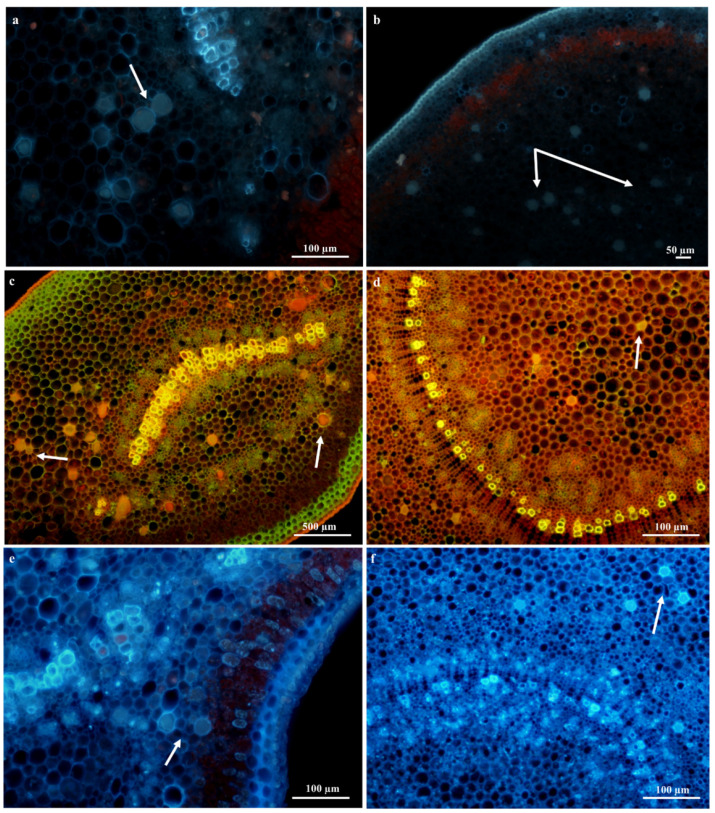
Fluorescence microscopy of young leaf midrib and young stem sections of *T. ventricosa*. (**a**) Auto-fluorescence of laticifer cell in leaf section showing intense blue fluorescence indicating the presence of phenolics. (**b**) Positive auto-fluorescence stain of stem section indicative of phenolics. (**c**,**d**) Leaf and stem sections stained orange-red using Acridine Orange indicating non-lignified laticifer contents. (**e**,**f**) Positive staining for cellulose using Calcofluor White on leaf and stem sections. Arrows refer to laticifer.

Preliminary observations with autofluorescence revealed the presence of plastids indicated by a red fluorescence and phenolics which displayed a high-intensity blue fluorescence color within the laticifer cells (latex) of vegetative organs (Figure 9a,b). Phenols have been reported to contain anti-oxidant, anti-inflammatory, and anti-bacterial properties [45,51]. A few of the cell walls of laticifers stained with Acridine orange in leaf sections (Figure 9c) displayed a yellow-green fluorescence; however, many laticifer cell walls in the leaf and stem sections (Figure 9c,d) also displayed a uniform red-orange coloration of high intensities. Lignified tissues are distinguished by a yellow-green fluorescence, whilst non-lignified tissue exhibits an orange-red fluorescence [52]. Calcofluor white staining depicted high concentrations of cellulose in laticifer walls [52], as these cells were observed to produce an intense blue color (Figure 9e,f). The composition of cell walls was examined in all histochemical and fluorescence tests to differentiate between neighboring cells and laticifers. These observations were based on cell thickness and composition of laticifer cell walls as standard measures [52]. 

### 2.5. Laticifer Ultrastructure of Adult Plant Leaves

**Figure 10:**
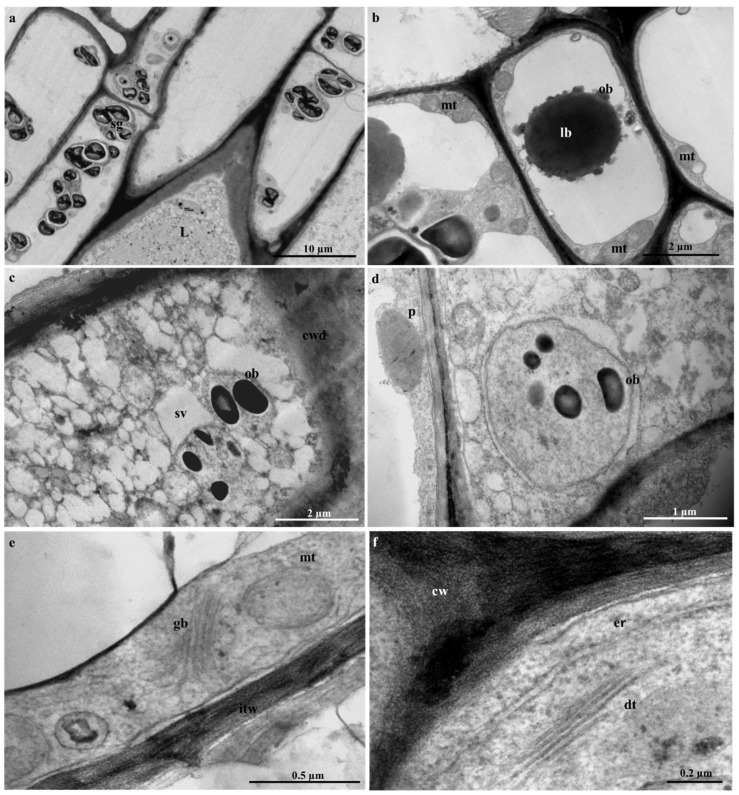
Ultrastructure of laticifers in the adult leaves of *T. ventricosa*. (**a**) Oblique section of a laticifer cell showing an acute apex and starch grains. (**b**) Lipid body closely associated with osmiophilic bodies. (**c**) Coalescence of small vacuole with granular laticifer cell content. (**d**) Osmiophilic bodies free in the cytosol and plastid with an electron-dense globule. (**e**) Presence of mitochondria, Golgi body and irregular thickening of cell walls. (**f**) Expansion of endoplasmic reticulum and presence of dictyosomes nearby the cell wall. Abbreviations: L = Laticifer, sg = starch grain, cwd = cell wall dissolution, sv = small vacuole, ob = osmiophilic bodies, p = plastid, gb = golgi body, mt = mitochondria, itw = irregular thickened walls, er = endoplasmic reticulum, dt = dictyosomes, cw = cell wall, lb = lipid body.

**Figure 11:**
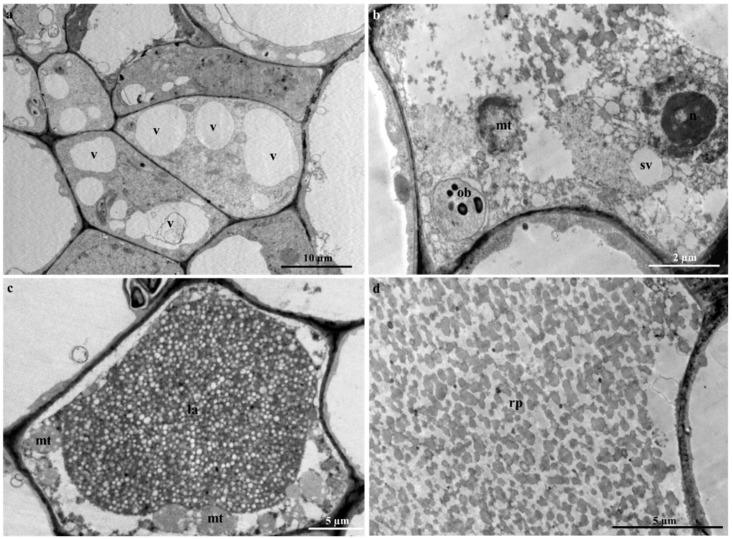
Latex production in adult leaves of *T. ventricosa*. (**a**) Highly vacuolated cytoplasm of young laticifer. (**b**) Initiation of secretory activity. (**c**) Latex metabolites forming an emulsion within the central vacuole of the laticifer cell. (**d**) Rubber particles within the latex emulsion. Abbreviations: v = vacuole, ob = osmiophilic bodies, mt = mitochondria, n = nucleus, sv = small vacuole, la = latex, rp = rubber particle.

Due to the difficulty experienced in the ultrastructural analyses of embryos, seedlings, and adult stems, only young leaf material was used for ultrastructural investigations. The laticifer system in the young leaves of *T. ventricosa* was formed by the accumulation of new cells, accompanied by a rapid discontinuity of transverse cell walls, resulting in the combination of their protoplasts (Figure 10). These ultrastructural changes have been similarly observed in other latex bearing species [18,31,33]. These cells were notable from surrounding ground and vascular tissue by contact with adjacent cells via cell wall dissolution (Figure 10c), irregularly thickened cell walls (Figure 10e), latex content (Figure 10c and Figure 11c,d) and their lengthened form (Figure 11a), similarly observed in other studies [18,31]. Irregularly shaped laticifers displaying acuminate ends (acute apex) were found appressed to the middle lamella of adjacent cells indicative of an oblique section of the apical cell as observed in Figure 10a [31]. With the analyses of the terminal walls of laticifers, it is possible to observe the expansion of laticifers via the dissolution of its cell walls and the concurrent accumulation of new cells to the existing laticifers, which has been observed in *T. catharinensis* (Figure 10a,c) [18,41]. The rapid cell wall dissolution of laticifers usually occurs from the center towards the periphery of the cell (Figure 10c) [41]. This process is achieved by small lytic vesicles formed from the peripheral endoplasmic reticulum [18,31,41]. Subsequently, the plasma membrane and tonoplast of both cells merge resulting in the formation of a continuous laticifer with one central vacuole [31,41].

Golgi bodies and dictyosomes were observed embedded within the cell (Figure 10e,f), with small vacuoles in proximity (Figure 11a). It has been suggested that these smaller vacuoles are the initial forms of a central vacuole and possibly contribute to the abundance of vesicles [18,31,53]. The formation of small vacuoles is due to the endoplasmic reticulum and electron dense material observed in the cytoplasm (Figure 10c). Initial subcellular alterations of the cytoplasm are due to the combination of tiny highly vacuolated cells (Figure 11a–c), that result in the establishment of a large central vacuole within a cell [18,31,41]. The increased size of the central vacuole was found to compress the cytoplasm into a thin parietal layer, likewise to literature [31]. At this stage of latex production, the contents of the cell are altered significantly [18,31], as an abundance of mitochondria (Figure 11b,c), lipid bodies (Figure 10b), osmiophilic bodies (Figure 10b–d and Figure 11b), plastids and starch grains are observed (Figure 10a,d). The occurrence of plentiful mitochondria is possibly associated with the supply and demand of energy required by the secretory process of the variable components of latex [31,54]. At the initiation of the secretory process, osmiophilic bodies are produced and relocated to the central vacuole. This process has been observed in *Calotropis gigantea* [55]. 

The latex that fills the laticifer lumen is regarded as the protoplast of the laticifer [41,56] and contains an emulsion of latex containing rubber particles within the parietal cytoplasm (Figure 11d). Furthermore, a rupture (natural or induced) of the laticifer cell wall results in the release of its latex contents, which is often used as protective mechanism by latex-bearing plants [26,27]. 

## 3. Materials and Methods

### 3.1. Collection of Leaf and Stem Samples

Fully grown and adult (~12–15 m) *T. ventricosa* plants of wild origin were collected from the University of KwaZulu-Natal (Westville campus), South Africa (1). 29°49′03.3″ S 30°56′32.7″ E; (2). 29°49′02.5″ S 30°56′32.5″ E; (3). 3.29°49′04.6″ S 30°56′43.9″ E, from February 2017 to October 2018. Approximately three plants of *T. ventricosa* were sampled for analysis, with the leaves and stems sampled in triplicates, for each plant. The plant material was taxonomically identified and a voucher specimen (18222) was deposited at the Ward herbarium, School of Life Sciences, University of KwaZulu-Natal. The leaves were classified into three different developmental stages namely, emergent (<10 mm), young (10–60 mm), and mature (>60 mm). For the purpose of stem analysis, only young stems were used, as mature stems were hardy and problematic for further examination. In addition, mature embryos and seedling stems of *T. ventricosa* were also studied. These specimens were acquired, by the collection of seeds from mature fruits of *T. ventricosa* growing in a sloped area (29°49′03.3″ S 30°56′32.7″ E) during 2017 and 2018. Seeds (*n* = 32) of *T. ventricosa* were carefully nicked (0.1 cm) using a sterile blade and soaked for approximately 48 h in distilled water [18]. Then, embryos were isolated from soaked seeds (*n* = 16). To obtain *T. ventricosa* seedlings, seeds (*n* = 16) were cultivated into trays containing vermiculite and maintained in a growth chamber under controlled light (12 h photoperiod) and air temperature (24 °C). The trays were watered regularly till seedlings emergence. After 20 days, the seedlings displayed two cotyledons, a stem (3 cm) and a main root (4 cm). 

### 3.2. Stereomicroscopy

Fresh emergent, young and mature leaf samples were used to analyze the adaxial and abaxial surfaces. Images were obtained using NIS-Elements D software on the Nikon AZ100 stereomicroscope equipped with a Nikon fiber Illuminator (Nikon, Tokyo, Japan). 

### 3.3. Scanning Electron Microscopy (SEM)

#### 3.3.1. Chemical Fixation

Young and mature leaves, and young stems of *T. ventricosa* were hand sectioned (±5 mm²) and fixed for 24 h in 2.5% glutaraldehyde. The sections were washed with 0.1 M phosphate buffer (pH 7.2) and post-fixed in 0.5% osmium tetroxide for 2 h. Thereafter, the samples were washed with 0.1 M phosphate buffer and subjected to dehydration by graded concentrations of ethanol (30%, 50%, 75%, and 100%). The sections were mounted onto aluminum, stubs using double-sided adhesive carbon tape and then critically point-dried using a Quorum K850 Critical Point Dryer, and sputter-coated with gold in a Quorum Q150 RES sputter coater. Samples were viewed with a scanning electron microscope Zeiss LEO 1450 at a working distance of 14–17 mm (5.00 kV). Images were captured using SmartSEM image software.

#### 3.3.2. Freeze-Fracture

Fresh young leaves and stems were trimmed (±5 mm²) and were quenched in liquid nitrogen slush (−210 °C), thereafter the sections were manually fractured using a blade. The fractured samples were freeze-dried in an Edward’s Modulyo EPTD3 freeze-drier for 72 h. The freeze-dried samples were mounted onto aluminum stubs using carbon adhesive tape and the stubs were coated with gold in a Quorum 150 RES sputter coater. The samples were then analyzed using Smart SEM imaging software on the LEO 1450 (Zeiss) scanning electron microscope at a working distance of 14–17 mm (5.00 kV).

### 3.4. Transmission Electron Microscopy (TEM)

Fresh young leaf and stem sections (adult plant), embryos, and the seedling stems (± 2mm²) were fixed in 2.5% glutaraldehyde for 24 h. The sections were washed with 0.1 M phosphate buffer (pH 7.2), followed by post-fixation with 0.5% osmium tetroxide for 4 h. Thereafter, the samples were washed with 0.1 M phosphate buffer and dehydrated sequentially in 30%, 50%, 75%, and 100% acetone. The dehydrated samples were infiltrated with 25%, 50%, and 75% of a Spurr’s resin and acetone mixture for 12 h, respectively. Thereafter, the samples were infiltrated in 100% resin for 24 h and then embedded in 100% resin using silicone molds and polymerized at 70 °C in an oven for 8 h. 

The resin block samples containing leaf and stem sections, embryos and seedling stems were sectioned using the Leica EM UC7 ultra-microtome equipped with a glass knife that was processed using a Glass Knife Maker LKB 7801A. Serial survey sections (0.5–1 µm) containing leaf and stem sections, embryos and seedling stems, respectively were placed onto slides, stained with Toluidine Blue and analyzed using NIS-Elements D imaging software on the Nikon Eclipse 80i light compound microscope (Nikon, Japan). The areas of interest were observed using serial survey sections, thereafter, ultrathin leaf sections (100 nm) were cut and picked up using copper grids. Prior to viewing, the copper grids were stained by placing the sections on a large drop of 2.5% uranyl acetate. The sections were stained for 10 min at 23 °C and rinsed with fresh distilled water. The copper grids were then placed onto drops of lead citrate in a closed glass Petri dish with dry NaOH pellets (avoids build-up of moisture which causes the stain to precipitate). The copper grids were stained for 10 min, rinsed with distilled water and placed on filter paper to dry. The sections were analyzed, and images were captured using a 100 kV JEOL 1010 transmission electron microscope equipped with iTEM software (JEOL, Peabody, MA, USA).

### 3.5. Histochemistry

Fresh young and mature leaves, and young stems were used to obtain semi-thin sections (80–100 μm) for histochemical analysis. The material was sectioned using the Oxford^®^ Vibratome sectioning system and the sections were stained with the following reagents to detect the presence and localization of chemical compounds. Toluidine Blue was used to test for the presence of carboxylated polysaccharides, phosphate groups, and polyphenols [57]. The presence of lipids was detected with Sudan IV and Sudan Black B [52]. Lignin aldehydes and cuticle components were detected with Phloroglucinol [52]. Phenolic compounds and terpenoids were tested using Ferric chloride and Ferric trichoride [52,58]. Ruthenium red was used to test for the presence of mucilage and pectin [52]. Nile blue was used to detect neutral and acidic lipids [52]. Proteins, essential oils, and resin acids were tested using Mercuric bromophenol blue [59] and NADI reagent [52], respectively, Alkaloids were detected using Wagner’s and Dittmar’s reagent [60]. Acidic components were detected using a double stain technique comprised of safranin and fast green [61]. Each test was carried out in triplicates (3 leaves and stems were sectioned, and 3 sections were used from each sample) and stained sections were analyzed using the NIS-Elements D imaging software and images were captured on the Nikon Eclipse 80i light equipped with Nikon DS-Fi1 compound microscope (Nikon, Japan). The presence of phenolic compounds was also tested using autofluorescence (330 and 380 nm) [62].

### 3.6. Fluorescence Microscopy

Semi-thin (80–100 μm) young leaf and stem sections were viewed at various wavelengths with a Nikon DS-Fi1 compound microscope (Nikon, Japan) equipped with NIS Elements D software. The sections were stained with Acridine orange and 0.01% Calcofluor white solution to detect the viability of cells and cellulose in cell walls, respectively [52]. 

### 3.7. Whole Mount Staining

Whole seedling leaves were fixed for 24 h at 4 °C in a solution of formalin, acetic acid and ethanol (3.5:10:50). The samples were washed with 70% ethanol and stained with Sudan Black B (0.1%) in 70% ethanol for 3–4 h at 23 °C. The samples were rinsed with 70% ethanol, distilled water and placed in 2.5 M NaOH solution until the leaves were cleared [33]. The samples were mounted onto a slide and the entire leaf was examined using the NIS-Elements D imaging software. Images were captured on the Nikon Eclipse 80i light compound microscope (Nikon, Japan).

## 4. Conclusions

The study concludes for the first time, the type and distribution of laticifers in the embryos, seedlings, and adult plants of *T. ventricosa*, and the plausible functions of laticifers and latex within this species. The ontogenetic studies of *Tabernaemontana ventricosa* confirmed the presence of articulated anastomosing laticifers. The laticifers were found to have originated from ground meristematic tissue and procambium cells, where they eventually dispense into all ground and vascular tissue. The complex branching patterns of laticifers usually develop into a composite system comprised of “Y”, “H”, or “U”conformations. The latter observations are consistent with literature, since recently majority of the laticifers belonging to the Apocynaceae are often classified as articulated. In addition, histochemical analyses revealed a variety of secondary metabolites including carboxylated polysaccharides, lipophilic and hydrophilic substances, proteins, phenolics, terpenoids, neutral lipids, alkaloids, mucilage, pectin, resin acids, and acidic substances present in the laticifers of *T. ventricosa.* Considering the chemical composition of the latex present in the lumen of laticifers, it is suggested that the latex is used as a protective mechanism against herbivory. Furthermore, the presence of alkaloids within the latex highlights its potential therapeutic value for the treatment of various ailments.
